# *Bifidobacterium* promotes retinal ganglion cell survival by regulating the balance of retinal glial cells

**DOI:** 10.1111/cns.14165

**Published:** 2023-03-16

**Authors:** Xiaohuan Zhao, Mengqiao Xu, Zhenzhen Zhao, Yimin Wang, Yang Liu, Ting Zhang, Xiaoling Wan, Mei Jiang, Xueting Luo, Yao Shen, Lei Chen, Minwen Zhou, Feng Wang, Xiaodong Sun

**Affiliations:** ^1^ Department of Ophthalmology, Shanghai General Hospital Shanghai Jiao Tong University School of Medicine Shanghai China; ^2^ National Clinical Research Center for Eye Diseases Shanghai China; ^3^ Shanghai Key Laboratory of Fundus Diseases Shanghai China; ^4^ Shanghai Engineering Center for Visual Science and Photomedicine Shanghai China; ^5^ Shanghai Institute of Immunology, Translational Medicine Center, Shanghai General Hospital Shanghai Jiao Tong University School of Medicine Shanghai China

**Keywords:** *Bifidobacterium*, microglia, Müller cells, optic nerve injury, retinal ganglion cells (RGCs)

## Abstract

**Introduction:**

Optic nerve injury is a leading cause of irreversible blindness worldwide. The retinal ganglion cells (RGCs) and their axons cannot be regenerated once damaged. Therefore, reducing RGC damage is crucial to prevent blindness. Accordingly, we aimed to investigate the potential influence of the gut microbiota on RGC survival, as well as the associated action mechanisms.

**Methods:**

We evaluated the effects of microbiota, specifically *Bifidobacterium*, on RGC. Optic nerve crush (ONC) was used as a model of optic nerve injury. Vancomycin and *Bifidobacterium* were orally administered to specific pathogen‐free (SPF) mice.

**Results:**

*Bifidobacterium* promoted RGC survival and optic nerve regeneration. The administration of *Bifidobacterium* inhibited microglia activation but promoted Müller cell activation, which was accompanied by the downregulation of inflammatory cytokines and upregulation of neurotrophic factors and retinal ERK/Fos signaling pathway activation.

**Conclusions:**

Our study demonstrates that *Bifidobacterium*‐induced changes in intestinal flora promote RGC survival. The protective effect of *Bifidobacterium* on RGC can be attributed to the inhibition of microglia activation and promotion of Müller cell activation and the secondary regulation of inflammatory and neurotrophic factors.

## Graphical abstract

*Bifidobacterium*‐induced changes of intestinal flora contributes to regulate retinal microglia and Müller cells activation, with alteration of inflammatory cytokines and neurotrophic factors.

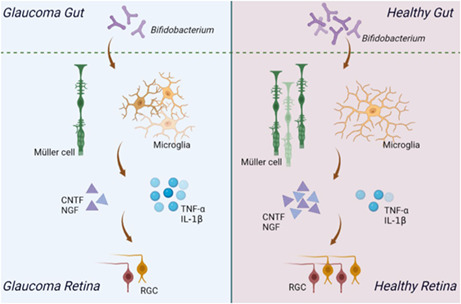

## 1 INTRODUCTION

Optic nerve injury, which can occur as a result of glaucoma, trauma, ischemia, optic neuritis, or optic nerve compression, is a major cause of irreversible blindness.^1^, ^2^ Globally, the number of patients with glaucoma is expected to increase to 111.8 million by 2040.^3^ Glaucoma is characterized by retinal ganglion cell (RGC) pathology, optic nerve degeneration, and progressive vision loss. As an extension of the central nervous system (CNS), the optic nerve and RGCs fail to regenerate once damaged.^4^ Therefore, reducing early neuron damage is crucial to preserve RGCs.

Retinal ganglion cell survival is regulated by several factors of the retinal microenvironment, including the interaction between the microglia and macroglia. Microglia reportedly aggravates RGC damage through the release of inflammatory cytokines, such as tumor necrosis factor α (TNF‐α), interleukin 1β (IL‐1β), and interleukin 6 (IL‐6).^5^, ^6^ Müller cells, a macroglia cell type, protect RGCs from glaucoma‐induced damage by secreting neurotrophic factors (ciliary neurotrophic factor (CNTF)) and antioxidants (glutathione and ghrelin).^7^, ^8^ We previously reported that in glaucoma, microglia prompt Müller cells to produce neurotrophic factors and help regulate the synaptic activity, and Müller cells assist microglia in regulating neuroinflammation in various ways.^9^

In a recent study, germ‐free (GF) mice presented with global microglia defects with altered cell proportions and an immature phenotype, which led to impaired innate immune responses.^10^ These results indicated that the gut microbiota regulates CNS neuroinflammation and neurotrophy. Furthermore, compared with specific pathogen‐free (SPF) mice, GF mice had lower levels of neurotrophic factors in the cortex and hippocampus.^11^ They are also closely related to several neurodegenerative diseases, including glaucoma. Patients with glaucoma have a higher oral microbial load^12^ and are prone to frequent *Helicobacter pylori* infections.^13^, ^14^ In addition, patients with primary open‐angle glaucoma have increased gram‐negative bacilli load.^15^ Notably, GF mice do not exhibit pathological manifestations such as RGC loss, even under the induction of high intraocular pressure,^16^ indicating that the gut microbiota plays an essential role in the pathogenesis of glaucoma. However, the effects of probiotics on RGC survival and regeneration have not been determined.

In this study, we aimed to determine the protective effects of intestinal flora, as well as its associated mechanisms, on RGCs. Notably, we investigated whether the intestinal flora, specifically *Bifidobacterium*, a gram‐positive anaerobic bacterium associated with neurodegenerative diseases,^17^, ^18^, ^19^ could promote RGC survival via retina glial cell (Müller cells and microglia) regulation.

## 2 METHODS

### 2.1 Animals

Female adult C57BL/6 mice, aged 6–8 weeks (body weight 15–18 g, Shanghai, China), were used. The water and food consumed by all mice were sterilized. Prior to all surgical procedures, the mice were injected intraperitoneally with sodium pentobarbital (50 mg/kg) as an anesthetic. Each experimental group consisted of 5–6 mice for the optic nerve crush (ONC). All animal experiments were approved by the Ethics Committee of Jiao Tong University, Shanghai, China, and were conducted in compliance with the Association for Research in Vision and Ophthalmology Statement for the Use of Animals in Ophthalmic and Vision Research.

### 2.2 Bacterial preparation

A lyophilized *Bifidobacterium* blend (Seeking Health, 12 billion CFU per capsule) was used, which included 7 *Bifidobacterium* strains: *Bifidobacterium* infantis (Bi‐26), *Bifidobacterium* bifidum/lactis (Bb‐02), *Bifidobacterium* longum (Bl‐05), *Bifidobacterium* bifidum (Bb‐06), *Bifidobacterium* lactis (Bl‐04), *Bifidobacterium* lactis (Bl‐07), and *Bifidobacterium* breve (Bb‐03). The blend was resuspended in sterilized phosphate buffer saline (PBS), according to previous research.^20^ *Bifidobacterium* (1 * 10^9^ CFU per mouse) was administered to each mouse via oral gavage. Heat inactivation was performed as previously described.^21^ Briefly, the rehydrated *Bifidobacterium* was boiled at 100°C for 2 h. Live and heat‐treated *Bifidobacterium* was diluted in PBS and plated on reduced clostridial medium (RCM) agar to incubate in an anaerobic chamber for 3 days to test the efficacy of killing.

For the ONC mouse, *Bifidobacterium* was given 3 days before ONC. The precise time is also indicated in the figure legends. A total of 300 μL *Lactobacillus rhamnosus LGG* (ATCC, 10^9^ PFU per mouse) or *Clostridium butyricum* (CB, Miyarisan Pharmaceutical Co., 10^9^ PFU per mouse) was orally administered to each mouse.

### 2.3 RNA isolation and RT‐PCR

Total RNA was isolated from the mouse retinas with a Trizol reagent according to the RNAsimple Total Kit instructions (Tiangen Biotech, Beijing, China), which was then reverse transcribed into complementary DNA (cDNA) with a reagent kit (Takara Bio Inc., Dalian, China). The RT‐PCRs, using a SYBR green‐based PCR method, were performed with a real‐time PCR detection system (Eppendorf, Hamburg, Germany). GAPDH was used as an internal reference to normalize the variation amounting to total cDNA. The forward and reverse primers for mouse GAPDH were 5′‐AGGTCGGTGTGAACGGATTTG‐3′ and 5′‐TGTAGACCATGTAGTTGAGGTCA‐3′. The forward and reverse primers for mouse iNOS were 5′‐GTTCTCAGCCCAACAATACAAGA‐3′ and 5′‐GTGGACGGGTCGATGTCAC‐3′. The forward and reverse primers for mouse TGF‐β were 5′‐CTCCCGTGGCTTCTAGTGC‐3′ and 5′‐GCCTTAGTTTGGACAGGATCTG‐3′. The forward and reverse primers for mouse Arg‐1 were 5′‐CTCCAAGCCAAAGTCCTTAGAG‐3′ and 5′‐AGGAGCTGTCATTAGGGACATC‐3′. The forward and reverse primers for mouse Egr1 were 5′‐TCGGCTCCTTTCCTCACTCA‐3′ and 5′‐CTCATAGGGTTGTTCGCTCGG‐3′; the forward and reverse primers for mouse Fos were 5′‐CGGGTTTCAACGCCGACTA‐3′ and 5′‐TTGGCACTAGAGACGGACAGA‐3′. Experiments were performed in three parallel wells and repeated at least twice.

### 2.4 Western blot analysis

A western blot analysis was used to measure GAP‐43, GFAP, c‐fos, ERK, and p‐ERK expression in the retinas of the *Bifidobacterium*‐treated mice. The retinas were collected and lysed. An equal amount of protein was added to 10% SDS‐PAGE gels, electrophoresed, and transferred electrophoretically onto polyvinylidene difluoride membranes. The membrane was blocked with a blocking buffer (Tris‐buffered saline Tween‐20 (TBST), containing 5% nonfat dry milk) for 1 h at room temperature and then incubated overnight with primary antibodies directed against GAP‐43 (Santa Cruz Biotechnology, 1:100), GFAP (Abcam, 1:1000), c‐fos (Abcam, 1:1000), ERK (CST, 1:1000), p‐ERK (CST, 1:1000), and GAPDH (Proteintech, 1:1000). The membranes were washed with TBST for 30 min and then incubated with a secondary antibody (Proteintech, 1:5000) for 1 h at room temperature. Proteins were visualized using a molecular imaging system (Amersham Imager 600, GE Healthcare, Buckinghamshire, UK). The experiments were repeated three times.

### 2.5 ELISA

Retinas were collected, weighted, and homogenized for the indicated time points. The levels of cytokines and neurotrophic factors in the retinas were detected with ELISA kits specific for mice, according to the manufacturer's procedures (CUSABIO). Results are expressed as picograms per milliliter of medium.

### 2.6 Immunofluorescence

Eyes on slides were permeated with 0.1% Triton X‐100 in PBS at room temperature for 20 min. Then, the slides were blocked (5% goat serum albumin (Beyotime)) for 1 h at room temperature, and tissues were incubated with primary antibodies against GFAP (Abcam, 1:1000), GS (Abcam, 1:1000), Tuj1 (Abcam, 1:1000), pS6 (Abcam, 1:1000), and c‐fos (Abcam, 1:1000) at 4°C overnight. The slides were infiltrated in PBS for 30 min, followed by an hour of incubation with secondary antibodies (Alexa Fluor 488 or Alexa Fluor 568 conjugated, Invitrogen, USA). DAPI was used to mark the nuclei. All slides were shot and analyzed with a Leica TCS 170 SP8 confocal laser scanning microscope (Leica TCS NT, Wetzlar, Germany).

### 2.7 Drug administration

Pexidartinib (PLX3397) was administered to pharmacologically ablate retinal microglia (290 mg/kg, MedChemExpress, HY‐16749). Vancomycin was administered on day 0 and lasted up to day 14; *Bifidobacterium* was administered on day 14; and ONC was performed on day 17. Mice were treated with PLX3397 from day 7 to day 31, and RGCs were observed on day 31.

### 2.8 Optic nerve crush

Optic nerve crush (ONC) was performed as previously described.^22^ First, the eyes were anesthetized with sodium pentobarbital by intraperitoneal injection (40 mg/kg). Then, a 0.5 cm skin incision was made with scissors at the lateral orbit. Blunt dissecting was performed to expose and isolate the optic nerve. The nerve was crushed using jeweler's forceps (Dumont #5, Roboz) for 10 seconds approximately 1 cm behind the optic disk. After ONC, the tissue around the incision was organized to allow the incision to close naturally. The retinas were analyzed at different times post injury (dpi): 1, 3, 7, 10, and 14 days. All operations were carried out by the same experienced researcher.

### 2.9 Quantification of *Bifidobacterium* by qPCR Assay

Fecal samples were collected 7 days after different antibiotics (ampicillin, colistin or vancomycin) treatment. Bacterial DNA in fecal samples was isolated by Qiagen DNA Stool Mini Kit according to the manufacturer's instructions.^23^ Taqman‐targeted qPCR was applied to quantify *Bifidobacterium*. Total bacterium was used to normalize the abundance of *Bifidobacterium*. The forward and reverse primers for total *Bifidobacterium* were CGGTGAATACGTTCCCGG and TACGGCTACCTTGTTACGACTT, and probe was 6FAM‐CTTGTACACACCGCCCGTC; the forward and reverse primers for *Bifidobacterium* were CGGGTGAGTAATGCGTGACC and TGATAGGACGCGACCCCA, and probe was 6FAM‐CTCCTGGAAACGGGTG.

### 2.10 Fecal transplantation

Donor mice were fed with vancomycin for 2 weeks, then gavaged with *Bifidobacterium*, and feces were collected 3 days later. The collected feces were treated and resuspended according to the method mentioned in a prior study^24^ and stored at −80°C. Recipient mice were also kept on vancomycin‐medicated water for 2 weeks and orally fed with transplanted feces (FMT).

### 2.11 Fecal bacteria analysis

The mice were given vancomycin orally for 2 weeks and then administered PBS, *Bifidobacterium*, or fecal suspensions via oral gavage. Feces were collected at 0, 3, 6, 10 and 17 days after gavaging, respectively. The feces of 6 mice were collected in each group (PBS, *Bifidobacterium*, and FMT). The collected feces were stored at −80°C for 16 s of sequencing. In brief, microbial deoxyribonucleic acid (DNA) was extracted using a PowerFecal Pro DNA Kit (QIAamp). Samples were quantified using the Qubit 1XdsDNA HS Kit (Thermo Fisher). PCR was used to amplify the sample. The concentration of the PCR products was detected using the Qubit 1XdsDNA HS Kit (Thermo Fisher). Then, 4 nM pool samples were prepared and purified using AMPure XP beads. The purified product was subjected to paired‐end sequencing using the Illumina MiSeq platform (Illumina, San Diego, U.S.A.). The meta‐sequence data were deposited at National Center of Biotechnology Information (NCBI) under Bioproject: PRJNA699743.

### 2.12 Bulk RNA‐Seq

Retinas were isolated and collected for RNA‐Seq at 0 and 3 days after the ONC of the three groups (PBS, *Bifidobacterium*, and FMT). The RNA‐Seq procedure was carried out as previously described.^25^

In brief, the mRNA enrichment method was used to determine the total RNA. Quality control for the raw data was used to determine whether the sequencing data were available for subsequent analysis. Then, total RNA was used as an input with the Illumina Tru‐Seq Library Preparation Kit v. 2 (Illumina, San Diego, CA, USA), and libraries were prepared based on the manufacturer's instructions. The sequencing was carried out using a BGIseq500 platform (BGI‐Shenzhen, China), and five biologic replicates for each type of sample were provided. Significant results were verified by RT‐PCR. The meta‐sequence data were deposited at NCBI under Bioproject: PRJNA698734.

### 2.13 Statistical analysis

Differences between groups were analyzed with a two‐tailed Student's *t*‐test, one‐way ANOVA, and two‐way ANOVA as appropriate for the specific experiment. The Shapiro–Wilk test and/or Kolmogorov–Smirnov test for normality were used to assess the data distribution. GraphPad Prism version 6.00 (GraphPad Software) was used for the analysis, and the results were presented as mean ± SD. A *p* value less than 0.05 was considered statistically significant (*p < 0.05, **p < 0.01, and ***p < 0.001).

## 3 RESULTS

### 3.1 Gram‐positive flora in mice protects RGCs

**FIGURE 1:**
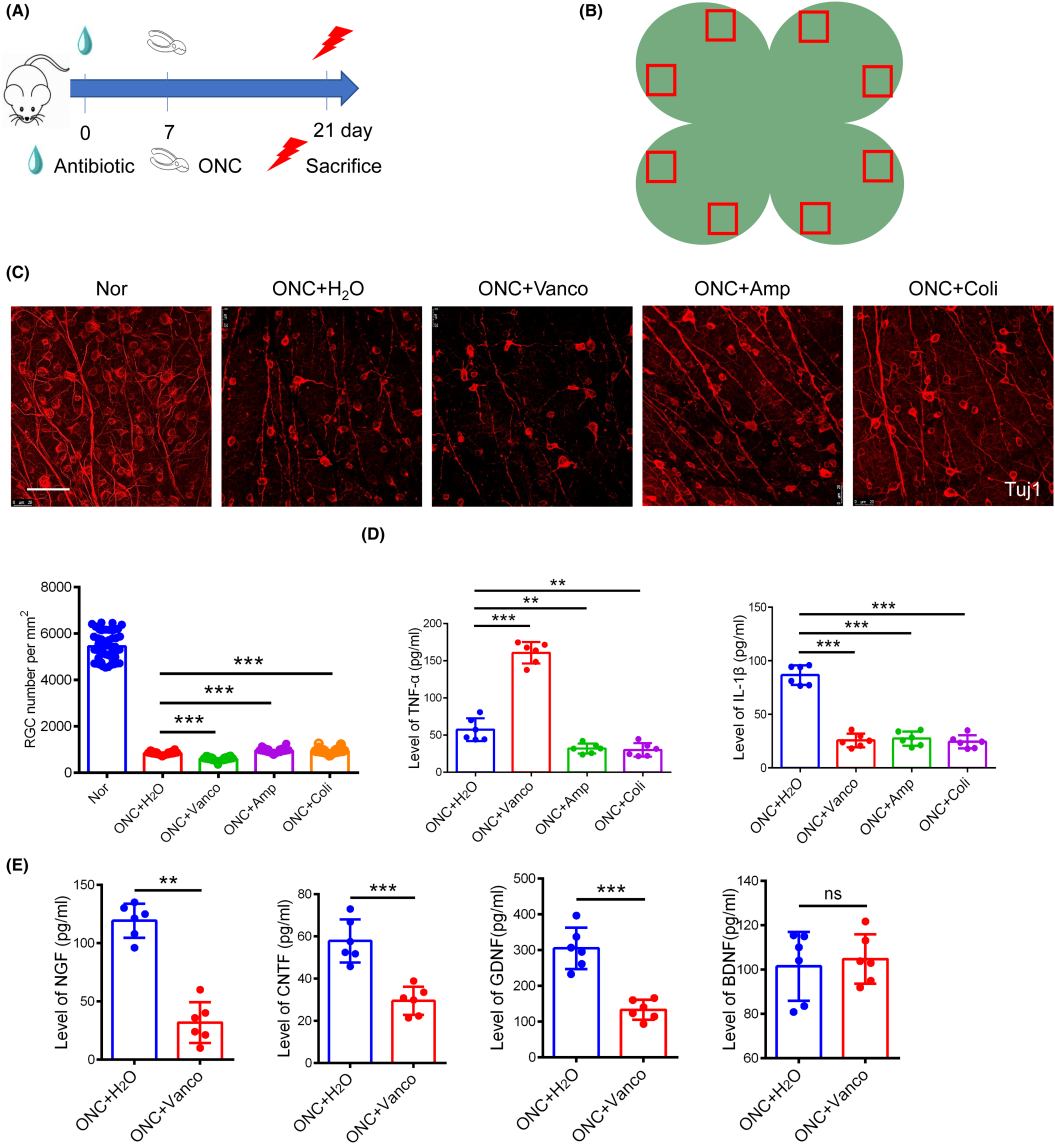
The resident intestinal flora of mice, especially the Gram‐positive flora, protects RGC. (A) The schedule for experimental procedures. Ampicillin(1 mg/mL), colistin (1 mg/mL) or vancomycin (0.5 mg/mL) was orally applied every day for 21 days; at the 7th day, mice were performed ONC; at the 21st day, mice were sacrificed and retina were collected. (B) The demonstration of the imaging areas of a whole retina. The red squares indicate the imaging area of the retina. (C) Representative immunofluorescence images of Tuj1 + RGCs and quantitative analysis on the numbers of surviving RGCs in different antibiotic intervention groups (*n* = 6, one‐way ANOVA). Nor: normal mice without ONC and antibiotic treatment. (D) Levels of inflammatory factors in retina after different antibiotic treatments by ELISA (*n* = 6, one‐way ANOVA). (E) Levels of neurotrophic factors in retina after vancomycin treatments by ELISA (*n* = 6, *t* test). Scale bar: 50 μm. Data represent mean ± SD. **p < 0.01, ***p < 0.001.

To explore the effects of gut microbiota on RGCs, mice were fed different antibiotics (ampicillin (1 mg/mL), colistin (1 mg/mL), or vancomycin (0.5 mg/mL)) for 21 days. On day 7 of antibiotic intervention, the mice were subjected to ONC, a classic model of optic nerve injury that induces RGC death.^22^ On day 21 of antibiotic intervention, the mice were sacrificed, and their retinas were isolated (Figure 1A,B). Retinal immunostaining for Tuj1 (Figure 1C), a marker of RGC axons, revealed that RGC survival increased after treatment with ampicillin (958.15 ± 92.91 vs. 843.27 ± 63.22 RGCs per mm^2^ (treatment vs. control), *p* < 0.05) and colistin (940.19 ± 106.90 vs. 843.27 ± 63.22 RGCs per mm^2^ (treatment vs. control), *p* < 0.05), which effectively act against gram‐negative bacteria. Notably, treatment with vancomycin (Figure 1C), which acts against gram‐positive bacteria, negatively impacted RGCs (598.98 ± 75.39 vs. 843.27 ± 63.22 RGCs per mm^2^ (treatment vs. control), *p* < 0.05), which suggests that gram‐positive bacteria may play a critical role in RGC survival.

We further evaluated the levels of inflammatory factors in the retina after treatment with different antibiotics. Compared with the control group, the expression of TNF‐α and IL‐1β in the retinas decreased after treatment with ampicillin or colistin, whereas vancomycin treatment increased TNF‐α and decreased IL‐1β levels (Figure 1D, *p* < 0.05). We evaluated changes in retinal neurotrophic factors and observed that vancomycin reduced the levels of CNTF, nerve growth factor (NGF), and glial cell‐derived neurotrophic factor (GDNF) after ONC (Figure 1E, *p* < 0.05). We found that the relative abundance of *Bifidobacterium* in vancomycin group decreased significantly, whereas it in ampicillin and colistin groups did not change significantly compared with control group (Figure S1).

### 3.2 *Bifidobacterium* promotes RGC survival and optic nerve regeneration

**FIGURE 2:**
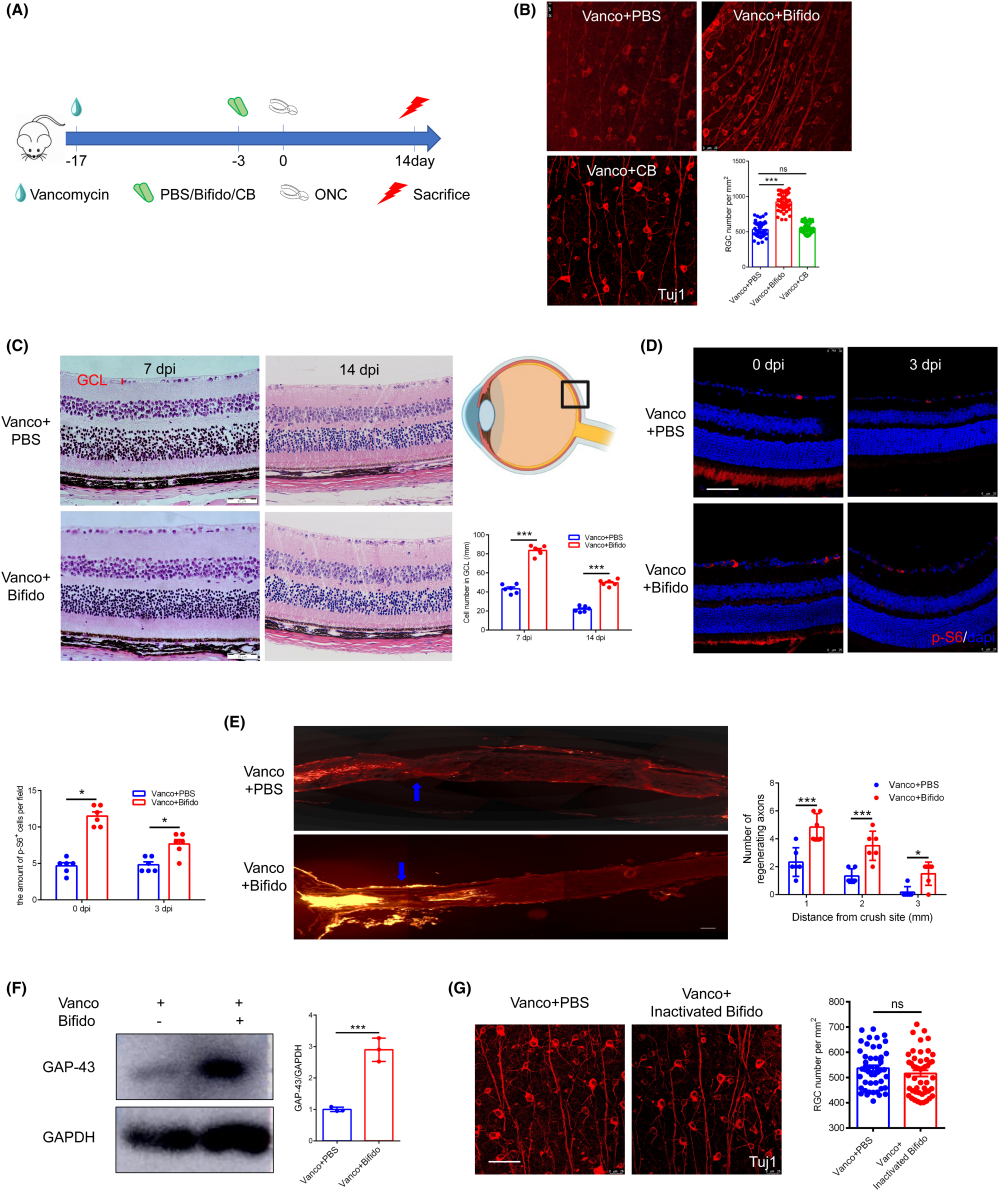
*Bifidobacterium* promotes RGCs survival and optic nerve regeneration. (A) The schedule for experimental procedures. Mice were orally given *Bifidobacterium* or CB after 2 weeks of vancomycin treatment, subjected to ONC 3 days after *Bifidobacterium* or CB treatment, and sacrificed to observe the number of RGCs 14 days after ONC. (B) Representative immunofluorescence images of Tuj1 + RGCs after *Bifidobacterium* or CB intervention at 14 dpi (*n* = 6, one‐way ANOVA). (C) The schematic diagram of section parts and representative HE images of retinal sections on cell numbers in GCL after *Bifidobacterium* treatment at 7 dpi and 14 dpi (*n* = 6, two‐way ANOVA). (D) Representative immunofluorescence images on the numbers of p‐S6+ cells after *Bifidobacterium* treatment at 0 dpi and 3 dpi (*n* = 6, two‐way ANOVA). (E) Optic nerve regeneration were traced by CTB after *Bifidobacterium* treatment at 14 dpi. The blue arrow indicates the site of crush (*n* = 6, two‐way ANOVA). (F) The expression of GAP‐43 protein at 7 dpi in PBS and *Bifidobacterium* group (*n* = 3, *t* test). (G) Immunofluorescence images of Tuj1 + RGCs after oral administration heat inactivated *Bifidobacterium* compared with PBS (*n* = 6, *t* test). Scale bar: 50 μm. For all graphs: error bars represent SD, *p < 0.05, ***p < 0.001.

To examine the effect of *Bifidobacterium* on RGC survival, *Bifidobacterium* was administered orally to mice after 2 weeks of vancomycin treatment. On day 3 of the *Bifidobacterium* treatment, mice were subjected to ONC. We evaluated the number of RGCs on day 14 of ONC (Figure 2A). Compared with the PBS group, oral administration of *Bifidobacterium* increased the number of Tuj1‐positive RGCs in the retina by 70% (Figure 2B; 531.71 ± 103.96 vs. 922.85 ± 122.02 per mm^2^, *p* < 0.05). No significant difference was observed between the number of RGCs in the CB and PBS groups (Figure 2B; 549.83 ± 79.09 vs. 531.71 ± 103.96 per mm^2^, *p* < 0.05). Approximately half of the cells in the ganglion cell layer (GCL) are RGCs.^26^ Therefore, we further observed the number of cells in the GCL of retinal slices at 7 and 14 dpi using hematoxylin–eosin (HE) staining (Figure 2C; 7 dpi, 83.52 ± 5.09 vs. 43.26 ± 4.63 per mm; 14 dpi, 49.72 ± 3.25 vs. 21.98 ± 3.03 per mm, *p* < 0.05). In the *Bifidobacterium* group, we found more viable cells in the GCL of the retinas.

To further confirm RGC survival, we measured phosphorylated S6 (p‐S6), a critical component downstream of mTOR signaling associated with RGC survival and axon regeneration.^27^ A larger number of GCL p‐S6‐positive cells were observed in the *Bifidobacterium* group before and after optic nerve injury (Figure 2D, *p* < 0.05).

We quantified optic nerve regeneration after *Bifidobacterium* treatment using cholera toxin subunit B (CTB)‐labeling. A higher amount of regenerated fibers were found in the *Bifidobacterium* group (Figure 2E, *p* < 0.05). In addition, compared with PBS treatment in the retina, *Bifidobacterium* promoted the expression of growth‐associated protein 43 (GAP‐43, Figure 2F), a crucial component of an effective regenerative response in the nervous system, up to three times (2.90 ± 0.37 vs. 1.00 ± 0.07, *p* < 0.05). Heat inactivation of *Bifidobacterium*, before oral administration, abrogated the RGC therapeutic effect on survival, which suggests that RGC protection requires live bacteria (Figure 2G; 516.00 ± 84.13 vs. 536.92 ± 78.35 RGC per mm^2^, *p* = 0.72).

### 3.3 Transfer of fecal microbiota promotes RGC survival

**FIGURE 3:**
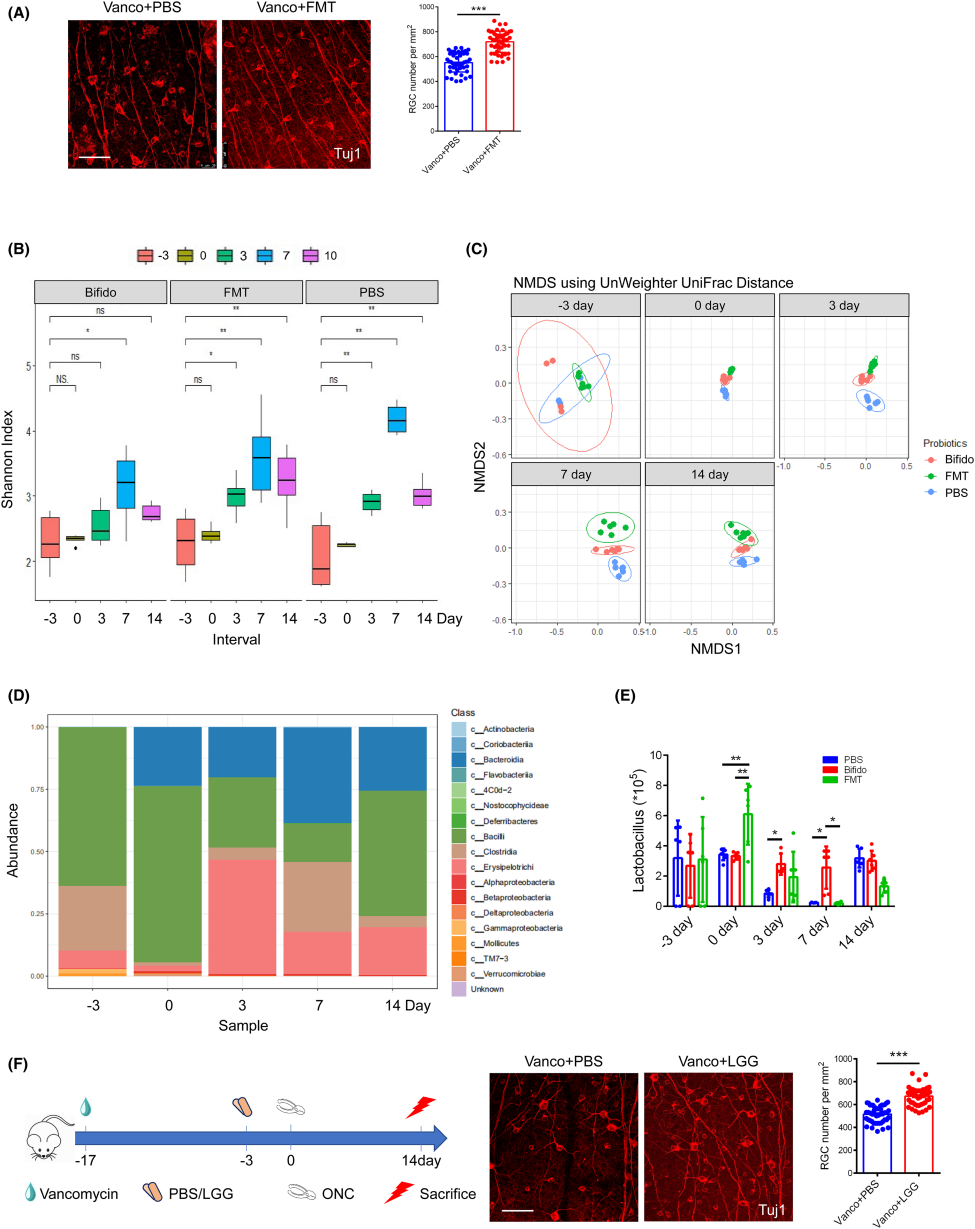
Transfer of fecal microbiota promotes RGCs survival. (A) Immunofluorescence images of Tuj1 + RGCs after FMT treatment at 14 dpi (*n* = 6, *t* test). (B, C) α‐Diversity (Shannon index) and β‐Diversity (UnWeighted Unifrac Distance) based on the genus profile at different time in the three groups. (D) Taxanomy barplot at class level based on the genus profile at different time. (E) Abundance of *Lactobacillus* in fecal material obtained from PBS, *Bifidobacterium* and FMT groups (*n* = 6, two‐way ANOVA). (F) The schedule for LGG‐treatment procedures and representative immunofluorescence images of Tuj1 + RGCs in PBS or LGG groups (*n* = 6, *t* test). Scale bar: 50 μm. Results are given as mean ± SD. *p < 0.05, **p < 0.01, ***p < 0.001 as compared to PBS group.

To clarify the implications of the altered gut microbiota on RGC survival, mice were treated with FMT and subjected to ONC. FMT increased RGC survival on day 14 post‐injury (Figure 3A, 719.50 ± 84.99 vs. 552.00 ± 76.30 RGC per mm^2^ (treatment vs. control), *p* < 0.05).

To determine the changes in intestinal microbiota after *Bifidobacterium* gavage, we performed 16S ribosomal RNA (rRNA) gene sequencing at −3, 0, 3, 7, and 14 dpi in PBS, *Bifidobacterium*, and FMT groups. *Bifidobacterium* and FMT administration resulted in significant fecal flora differences. Specifically, there were significant differences in α‐diversity at different times between the three groups (Figure 3B and Figure S2A). In addition, β‐diversity was similar at −3 dpi and gradually divided into three clusters (Figure 3C and Figure S2B). Over time, the species composition of the fecal flora changed (Figure 3D). LEfSe and linear discriminant analysis (LDA) scores were used to analyze the differences in the dominant bacterial distribution in the PBS, *Bifidobacterium*, and FMT groups (Figure S3A,B). An increase in *Lactobacilli* was observed in mice intestines after oral administration of *Bifidobacterium* and FMT, which persisted for at least 10 days following gavage (Figure 3E, *p* < 0.05). *Lactobacilli* increases were first observed in intestines following FMT treatment but persisted for a shorter period. We observed that oral administration of *Lactobacilli* to mice increased the number of RGCs by 30% (Figure 3F, 673.35 ± 78.56 vs. 516.15 ± 72.59 RGCs per mm^2^ (treatment vs. control), *p* < 0.05).

### 3.4 *Bifidobacterium* inhibits microglia activation and reduces inflammatory cytokines

**FIGURE 4:**
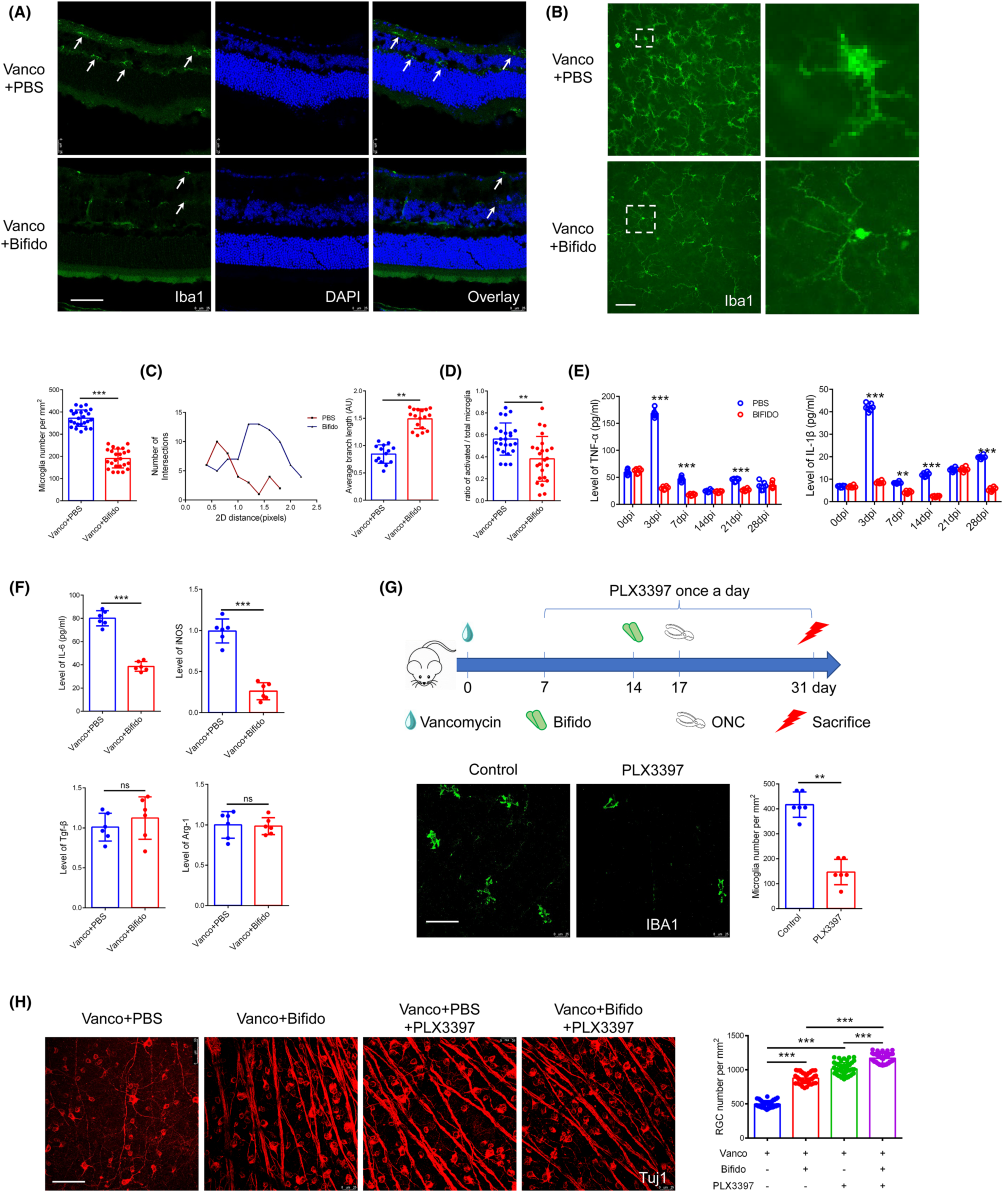
*Bifidobacterium* inhibits the activation of microglia and reduces the level of inflammatory cytokines. (A) Immunofluorescence images of Iba1+ in retinal sections after *Bifidobacterium* treatment at 3 dpi. (B) Immunofluorescence images of Iba1+ in retinal whole mount after *Bifidobacterium* treatment at 3 dpi (*n* = 6, *t* test). (C) Sholl analysis and Skeleton analysis were conducted to quantify microglia morphology (*n* = 6, *t* test). The less interaction number is, the more activated microglia is. Similarly, the less average branch length is, the more activated microglia is. (D) The ratio of activated/total microglia according to morphology (*n* = 6, *t* test). (E) Levels of inflammatory factors in retina at different time after *Bifidobacterium* treatment by ELISA (*n* = 6, two‐way ANOVA). (F) Levels of subtype markers of microglia in retina at 3 dpi after *Bifidobacterium* treatment (IL‐6 was evaluated by ELISA, and iNOS, Tgf‐β and Arg‐1 was tested by RT‐PCR, *n* = 6, *t* test). (G) The schedule for PLX3397‐treatment procedures and immunofluorescence images of Iba1+ microglia after PLX3397 treatment (*n* = 6, *t* test). (H) Immunofluorescence images of Tuj1+ RGCs after PLX3397 and *Bifidobacterium* treatment (*n* = 6, one‐way ANOVA). Scale bar: 50 μm. For all graphs: error bars represent SD, *p < 0.05, **p < 0.01, ***p < 0.001.

We examined microglia activation and cell number in the retina following *Bifidobacterium* treatment. In the *Bifidobacterium* group, retinal slices were examined at 3 dpi, and fewer microglia cells were observed in the GCL and outer plex layer (OPL) (Figure 4A). Microglia cell number and morphology were determined using stretched preparations and immunofluorescence at 3 dpi. Compared with the PBS control group, fewer microglia cells were observed in the *Bifidobacterium* group (Figure 4B; 190.32 ± 42.53 vs. 372.19 ± 36.90 RCGs per mm^2^, *p* < 0.05). In the *Bifidobacterium* group, microglia were long and extended with more branches, whereas microglia in the PBS retinas were shrunken (Figure 4B). Sholl and quantitative skeleton analysis revealed a higher number of intersections and longer branches, respectively, in the *Bifidobacterium* group (Figure 4C, 1.49 ± 0.18 vs. 0.84 ± 0.17 AU, *p* < 0.05). Compared with PBS group, a lower rate of microglia was activated in *Bifidobacterium* group (Figure 4D, 0.56 ± 0.15 vs 0.38 ± 0.20, *p* < 0.05). These results indicate that *Bifidobacterium* contributes to microglia activation.

Inflammatory cytokines, TNF‐α and IL‐1β, were evaluated at different time points after ONC. Following ONC, the levels of TNF‐α and IL‐1β in the PBS control group were higher than that observed in the *Bifidobacterium* group (Figure 4E, *p* < 0.05). Specifically, TNF‐α in the *Bifidobacterium* group was 17% of that in the PBS group, and IL‐1β in the *Bifidobacterium* group was 19% of that in the PBS group, at 3 dpi. In addition, the levels of IL‐6 and inducible nitric oxide synthase (iNOS, a marker of M1 microglia) were lower at 3 dpi in the *Bifidobacterium* group. No significant difference was observed in the expression of M2 microglia markers, including transforming growth factor β (TGF‐β) and arginase 1 (Arg‐1), between the PBS and *Bifidobacterium* groups (Figure 4F, *p* < 0.05).

To verify that *Bifidobacterium* protects RGCs via regulation of microglial cell number and activation, mice were treated with PLX3397 by oral administration. As an inhibitor of the CSF1R receptor, PLX3397 reduces tissue macrophages without affecting myeloid cells.^28^ PLX3397 eliminates mononuclear phagocytes, including microglia. We started gavage on day 7, and the dose was administered daily until day 31. Vancomycin was administered from days 0–14, and *Bifidobacterium* was administered on day 14 (Figure 4G). The ONC model experiment was conducted on day 17, and the RGCs were observed on day 31. PLX3397 gavage significantly reduced the number of retinal IBA1‐positive microglia cells (Figure 4G; 146.40 ± 50.86 vs. 416.67 ± 50.86 per mm^2^, *p* < 0.01). We further monitored RGC numbers following PLX3397 and *Bifidobacterium* treatment and observed that RGC survival increased in PBS and *Bifidobacterium* groups after PLX3799 administration (Figure 4H, *p* < 0.01). However, the number of RGCs in the *Bifidobacterium* group was higher than that in the PBS group.

### 3.5 *Bifidobacterium* promotes the secretion of CNTF and the activation of Müller cells

**FIGURE 5:**
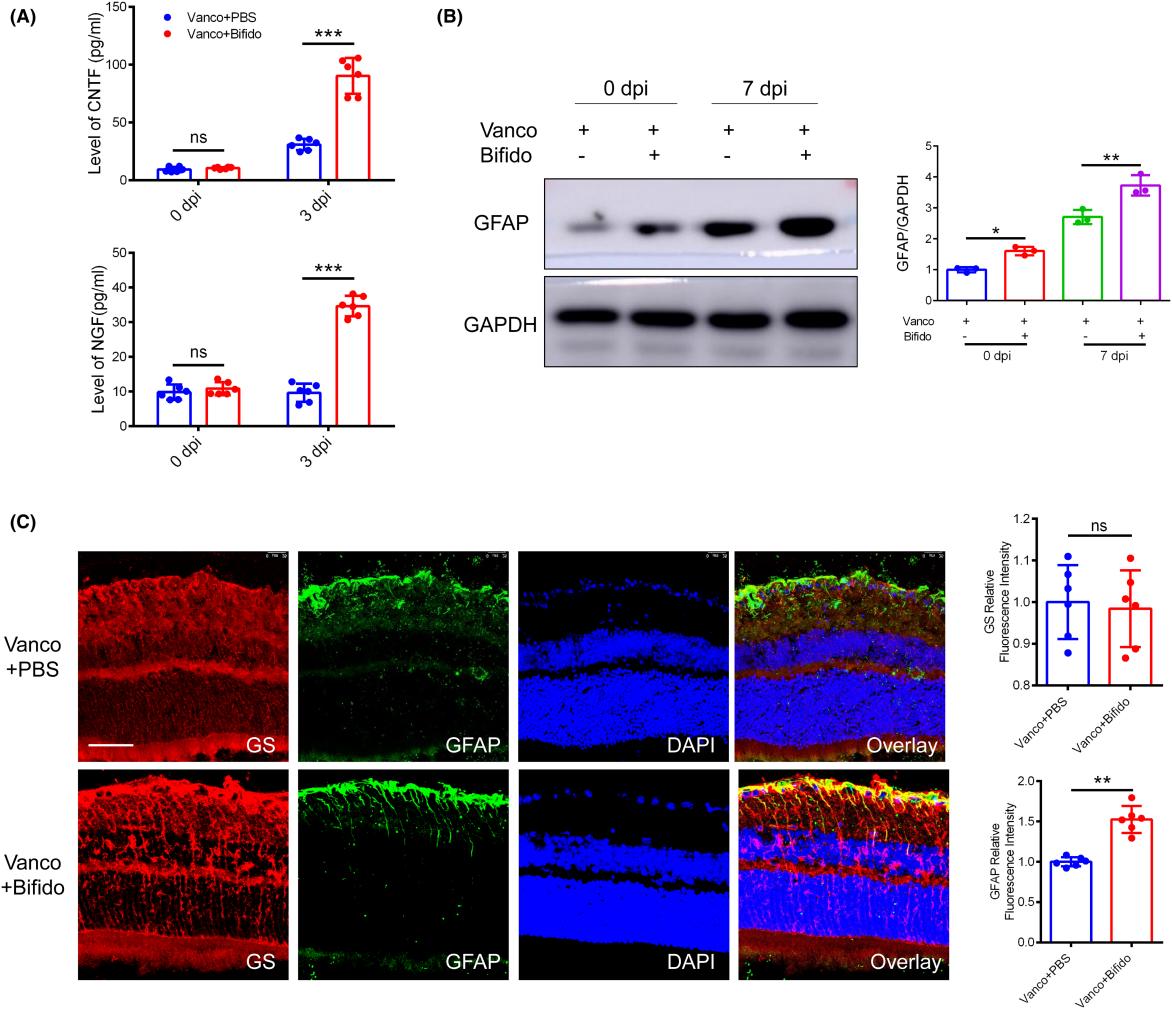
*Bifidobacterium* promotes the secretion of CNTF and the activation of Müller cells. (A) Levels of neurotrophic factors in retina after *Bifidobacterium* treatment by ELISA (*n* = 6, two‐way ANOVA). (B) The expression of GFAP protein at 0 dpi and 7 dpi in PBS and *Bifidobacterium* group (*n* = 3, two‐way ANOVA). (C) The expression of GFAP and GS at 7 dpi after ONC by immunofluorescence (*n* = 6, *t* test). Scale bar: 25 μm. For all graphs: error bars represent SD, *p < 0.05, **p < 0.01, ***p < 0.001.

Changes in intestinal microbiota alter the hippocampal expression of neurotrophic factors.^29^ To determine whether *Bifidobacterium* affects the production of neurotrophic factors in the retina, we used qPCR to quantify the levels of CNTF and NGF in mouse retinas at days 0 and 3 following ONC. The expression levels of CNTF and NGF in the *Bifidobacterium* group were higher than those in the PBS group 3 days following ONC (Figure 5A, *p* < 0.05), which indicates that *Bifidobacterium* increases the expression of neurotrophic factors.

In neurodegenerative diseases such as glaucoma, activated Müller cells produce CNTF and NGF.^30^, ^31^, ^32^ Thus, we used immunofluorescence and western blot analyses to measure the expression of the glial fibrillary acidic protein (GFAP), a glial cell marker. We observed that GFAP expression increased significantly on day 7 in the *Bifidobacterium* group (Figure 5B, *p* < 0.05). Notably, on day 0, when undamaged, the *Bifidobacterium* group also showed increased GFAP expression. CNTF or NGF expression did not differ significantly between the groups (Figure 5A). *Bifidobacterium* administration induced Müller cell activation and increased the expression of neurotrophic factors, CNTF or NGF. However, there was no significant difference in glutamine synthetase (GS) expression between groups (Figure 5C, *p* < 0.05).^33^ GS regulates extracellular glutamate levels and specifies the number of Müller cells. Therefore, the results indicate that *Bifidobacterium* induces Müller cell activation and elevates the production of neurotrophic factors but does not increase the number of Müller cells.

### 3.6 Oral administration of *Bifidobacterium* activates the retina ERK/Fos signaling pathway

**FIGURE 6:**
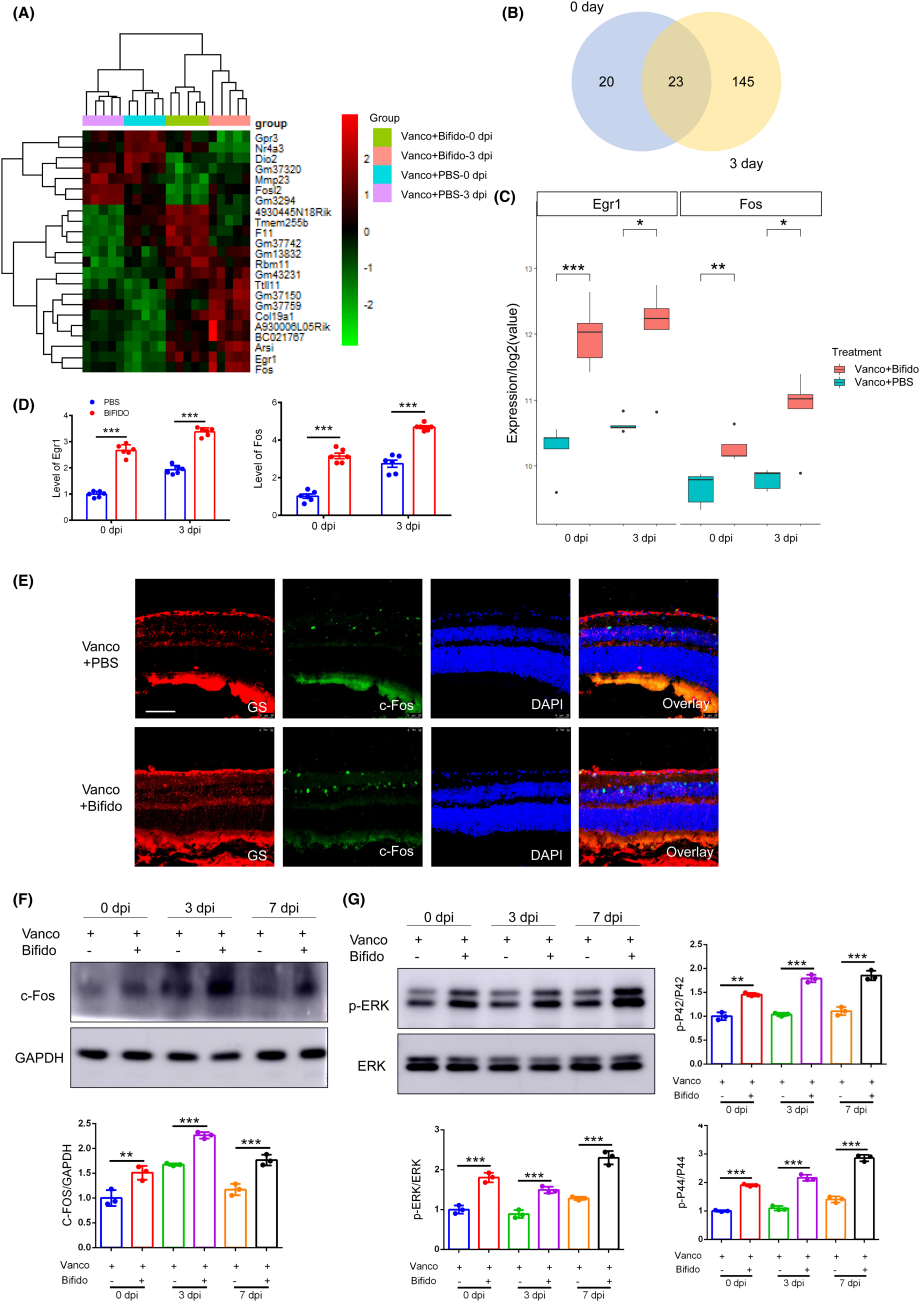
Oral administration of *Bifidobacterium* activates retina ERK/Fos signaling pathway. (A) Heat map of retinal transcriptome after *Bifidobacterium* administration before and after ONC (*n* = 5 per group). (B) The number of differential expressed genes and their intersection between *Bifidobacterium* and PBS groups at 0 and 3 days after ONC. (C) The mRNA expression of Fos and Egr‐1 according to RNA‐seq. (D) The mRNA expression of Fos and Egr‐1 by qPCR (*n* = 6, two‐way ANOVA). (E) The location of c‐Fos by immunofluorescence at 3 dpi. (F) The protein expression of c‐Fos in PBS and *Bifidobacterium* group by Western blot (*n* = 3, two‐way ANOVA). (G) The protein expression of ERK and p‐ERK in PBS and *Bifidobacterium* group by Western blot (*n* = 3, two‐way ANOVA). Scale bar: 50 μm. For all graphs: error bars represent SD, *p < 0.05, **p < 0.01, ***p < 0.001.

To further elucidate the mechanism by which *Bifidobacterium* regulates the retinal microenvironment, we determined differentially expressed genes in mouse retinas using bulk RNA sequencing (RNA‐seq). In the absence of injury (0 dpi group) and on day 3 following ONC (3 dpi group), there were gene expression differences in the retinal transcriptome between the PBS and *Bifidobacterium* groups (Figure 6A). We identified 23 overlapping genes, with varying expression levels, at 0 and 3 dpi (Figure 6B). In particular, the mRNA expression of early growth response protein 1 (*Egr1*) and *Fos* were higher in the *Bifidobacterium* group than in the PBS group (Figure 6C, *p* < 0.05). These results were further verified using qPCR analysis (Figure 6D, *p* < 0.05). *Egr1*, an immediate early gene, responds to stress, such as injury, and participates in cell proliferation and other activities. The *Fos* gene encodes proteins that dimerize with c‐Jun to form a transcription factor complex activator protein‐1 (AP‐1) heterodimer, which regulates cell proliferation, differentiation, and transformation. Notably, Egr1 and Fos are associated with retinal Müller cell activation. We further measured c‐Fos expression using immunofluorescence and western blotting (Figure 6E,F). Immunofluorescence revealed that c‐Fos was dominantly expressed in the INL, where glial nuclei are concentrated. Oral administration of *Bifidobacterium* increased the expression of the Fos protein in the INL (Figure 6F, *p* < 0.05). As a downstream effector of some intracellular signaling pathways, ERK/c‐Fos signaling mediates Müller cell activation and macrophage differentiation.^34^, ^35^, ^36^ To explore whether ERK/c‐Fos signaling in the retina was activated by *Bifidobacterium*, we measured the expression of total ERK and phosphorylated ERK (p‐ERK) at days 0, 3, and 7 post‐ONC in the PBS and *Bifidobacterium* groups (Figure 6G, *p* < 0.05). There was no significant difference in total ERK expression between the groups. The ratio of phosphor‐p42/total p42 to phosphor‐p44/total p44 in the *Bifidobacterium* group was significantly higher than that in the PBS group at each time point, which was consistent with c‐Fos expression. This indicates that *Bifidobacterium* triggers the ERK/c‐Fos signaling pathway.

## 4 DISCUSSION

**FIGURE 7:**
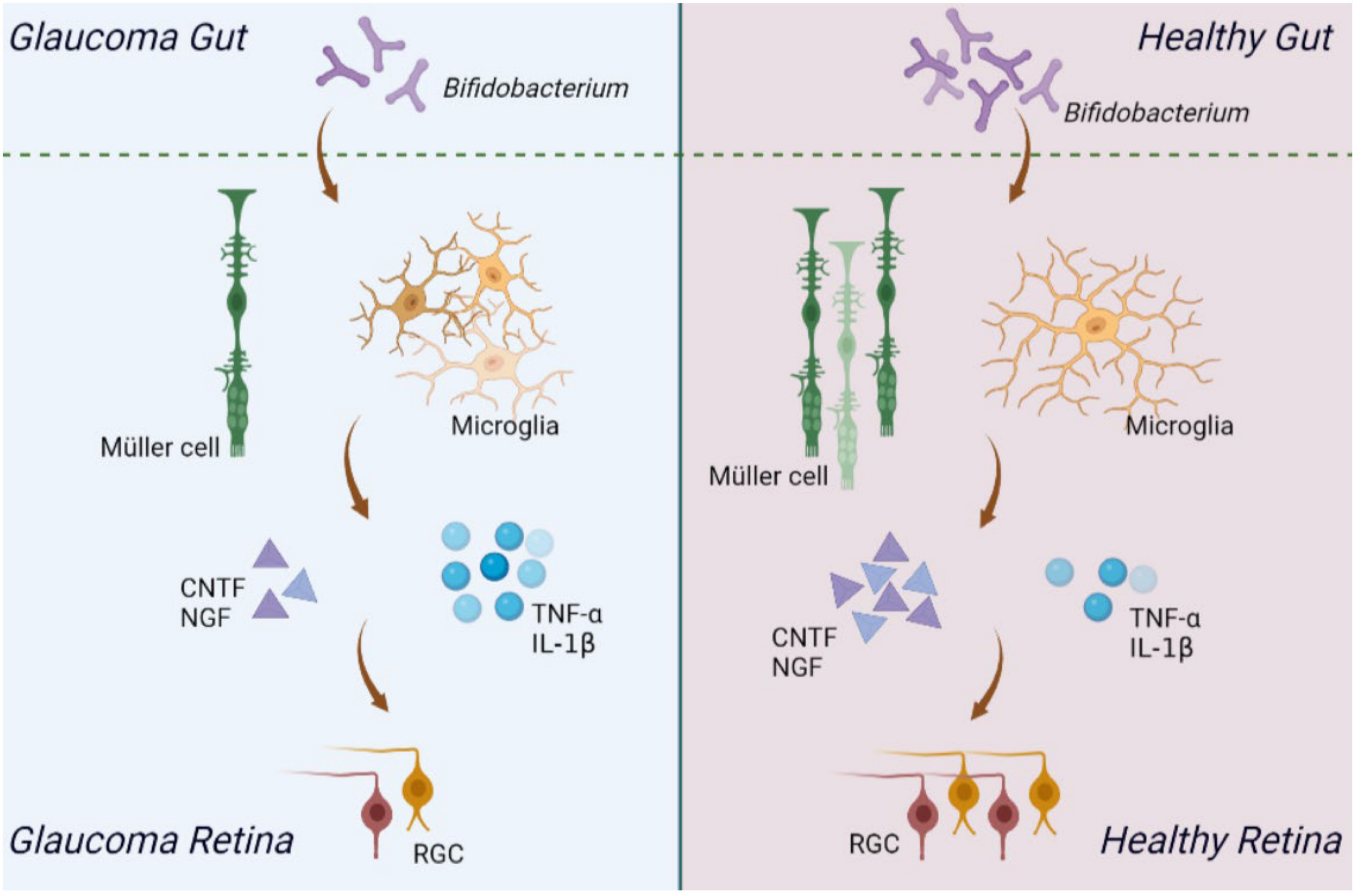
*Bifidobacterium* promotes RGCs survival by regulating the balance of retinal glial cells.

The objective of this study was to investigate the potential influence of gut microbiota on RGC survival. We demonstrated that gram‐positive flora, specifically *Bifidobacterium*, protects RGC survival and optic nerve regeneration. *Bifidobacterium*‐induced changes in the intestinal flora contribute to retinal microglia regulation and Müller cell activation, which leads to modifications in inflammatory cytokines and neurotrophic factors (Figure 7). Thus, our findings support the hypothesis that gut–retina communication affects the development of glaucoma neuropathology.

GF mice with high intraocular pressure are protected from loss of RGCs, suggesting that gut microbes play an indispensable role in glaucoma progression.^16^ In this study, eliminating intestinal microorganisms with vancomycin reduced the number of RGCs following ONC, whereas ampicillin and colistin increased the number of RGCs. Antibiotics with little or no oral bioavailability have been used to ablate a wide range of bacteria, specifically in the intestine. Vancomycin is not absorbed orally, colistin is absorbed with low oral bioavailability,^37^ and ampicillin is absorbed from the human gut. However, the concentration of ampicillin in the brain falls below the lower limit of detection.^38^ Thus, these three antibiotics are unlikely to be directly absorbed by the retina or directly regulate the number of RGCs. Vancomycin predominantly acts against gram‐positive bacteria, whereas ampicillin and colistin are mainly used against gram‐negative bacteria. Lipopolysaccharides, a component of the gram‐negative bacteria cell wall, aggravates glaucoma,^12^ which is consistent with the findings of this current study; that is, RGCs are protected by antibiotics that fight against gram‐negative bacteria. Vancomycin is reportedly non‐toxic to the retina.^39^, ^40^ Therefore, we hypothesized that gram‐positive bacteria were the main bacteria in the intestinal flora that played a protective role in RGCs. This is consistent with another study that reported a substantially higher amount of gram‐negative Bacilli in fecal samples of patients with primary open‐angle glaucoma.^15^

Supplementation with *Bifidobacterium* reversed the vancomycin‐induced loss of RGCs in mice, suggesting that *Bifidobacterium* is a key bacterial group that promotes the survival of RGCs. Additionally, the administration of *Bifidobacterium* contributed to optic nerve regeneration. The RGC protective ability of *Bifidobacterium* is dependent on live bacteria. *Bifidobacterium* promotes RGC survival through secondary changes in the composition of gut microbiota, including elevating levels of *Lactobacillus*. The necessity for live bacteria may imply that *Bifidobacterium* colonizes a specific chamber within the gut that enables it to interact with other bacteria, such as *Lactobacilli*. The relationship between glaucoma and microorganisms is well documented; however, the detailed mechanisms and specific functional strains are unclear. Our study has contributed to the understanding of the role of bacteria in glaucoma by demonstrating that gram‐positive bacteria, specifically *Bifidobacterium* and *Lactobacillus*, are beneficial to RGCs. Thus, the gut microbiota could serve as a potential target for the clinical prevention and treatment of glaucoma. Further research is required to confirm its clinical significance.

We also observed that the administration of *Bifidobacterium* inhibited microglia cell number and activation but promoted Müller cell activation, and this process was accompanied by the downregulation of inflammatory cytokines and the upregulation of neurotrophic factors. A recent study on Alzheimer's disease (AD) found that *Bifidobacterium breve MCC1274* attenuated microglial activation, consequently leading to a downregulation of pro‐inflammatory cytokines.^41^ Furthermore, *Bifidobacterium longum CCFM1077* ameliorates microglia activity in the cerebellum of autistic‐like rats.^42^ In addition, *Lactobacillus reuteri NK33* and *Bifidobacterium adolescentis NK98* significantly suppressed the infiltration of activated microglia into the hippocampus and corticosterone in mice, and hippocampal BDNF expression was also evaluated.^43^ GF mice have lower levels of neurotrophic factors in the cortex,^11^ and *Bifidobacterium longum* supplementation improves neurotrophic factor expression in AD.^44^ *Lactobacillus* was significantly depleted in the buccal microbiome of patients with uveitic glaucoma, which is associated with a broad spectrum of inflammatory diseases.^45^, ^46^ *Lactobacillus* ameliorates chronic inflammation by decreasing TNF‐α expression in the gut mucosa.^47^ These studies indicate that *Bifidobacterium* and *Lactobacillus* modulate the activity of microglia and the levels of inflammatory cytokines and neurotrophic factors in the CNS. In addition, we observed that the retina, an extension of the CNS, is similarly affected by *Bifidobacterium* and *Lactobacillus*. Furthermore, in our review of macroglia–microglia interactions in glaucoma, we reported that a delicate balance of glia is critical for the survival of RGCs.^9^ Therefore, *Bifidobacterium*‐induced intestinal changes likely play a role in protecting RGCs and promoting optic nerve regeneration by regulating the activity of glial cells, neurotrophic factors, and inflammatory factors in the retina.

We propose that *Bifidobacterium* triggers ERK/c‐Fos signaling pathways independent of ONC. We observed that the expression of p‐ERK and c‐Fos increased after *Bifidobacterium* treatment, regardless of optic nerve damage. The ERK/c‐Fos pathway may mediate the activation of Müller glial cells in retinal pathology.^48^, ^49^ The secretion of neurotrophic factors in the cerebral cortex is regulated by the ERK/c‐Fos signaling pathway,^48^ and the neuroprotective effect of estrogen on ONC‐induced RGC death occurs through activation of the ERK/c‐Fos signaling pathway.^49^ Macrophage differentiation, histologically similar to microglia, is thought to be regulated by ERK/c‐Fos signaling, with increased ERK/c‐Fos‐inducing macrophages differentiating into the anti‐inflammatory M2 type.^36^ We observed that c‐Fos was highly expressed after *Bifidobacterium* treatment and was primarily distributed in the INL, where glial nuclei were concentrated. Therefore, *Bifidobacterium* likely activates the ERK/c‐Fos signaling pathway in glial cells, regulates glial cell activity, and promotes RGC survival.

The anti‐inflammatory and neuroprotective effects of *Bifidobacterium* are not limited to glaucoma but may offer protection in many similar neurodegenerative diseases. The protective effect of *Bifidobacterium* has been mentioned in studies involving other neurodegenerative diseases,^50^ such as AD. Specifically, fewer *Bifidobacterium* species were found in the feces of patients with AD than in those of healthy individuals.^51^ In AD animal models, *Bifidobacterium longum* improved cognitive function and promoted BDNF expression.^44^ Findings from a randomized, double‐blind clinical trial suggested that probiotics, including *Bifidobacterium*, can significantly improve cognitive function in patients with AD.^52^ Therefore, our research has broadened the understanding of the action of *Bifidobacterium* as a well‐known probiotic, and the mechanisms we have proposed here can serve as the basis for further study of *Bifidobacterium* and neurodegenerative diseases. Overall, the application of appropriate probiotics is expected to become a safe and effective neurotrophic drug based on their role in promoting neurotrophic factors in neurodegenerative diseases.

There are some potential limitations in this study. First of all, the exploration of the key connection between gut and retina is still lacking. Currently, it is believed that the possible pathways of communication between gut microbes and retina include: the production of neuroactive metabolites, short‐chain fatty acids and other microbial products, nerves (intestinal nerves and vagus nerves), hypothalamic–pituitary–adrenal axis, endocrine (intestinal hormones), and immunity (immune cells and cytokines) etc. However, we have not yet explored the possible underlying mechanism of protective effects of *Bifidobacterium* on RGCs. Secondly, the ONC model used in this study is essentially an optic nerve injury model, which may not fully characterize the pathogenesis of glaucoma in human. It is generally believed that intraocular pressure is one of the most important factors in the pathogenesis of glaucoma. While ONC model is independent of intraocular pressure, thus this finding could only apply to RGC injuries independent of ocular hypertension in the clinical setting. Although we have revealed the important role of *Bifidobacterium* in the pathogenesis of RGC loss, future studies are critical to identify the comprehensive mechanisms connecting *Bifidobacterium* in gut and RGCs in the retina.

In conclusion, our data suggest that RGC survival in mice is mediated by the intestinal microbiota, specifically, *Bifidobacterium*‐induced changes. The protective effect of *Bifidobacterium* on RGC could be attributed to the balance between microglia and Müller cells, triggering the ERK/c‐Fos signaling pathways in the retina. Further investigation is required to determine the in‐depth protective mechanism and could illuminate the broader therapeutic potential of *Bifidobacterium* in the clinical treatment of neurogenerative diseases.

## AUTHOR CONTRIBUTIONS

Xiaohuan Zhao, Mengqiao Xu and Zhenzhen Zhao conceived the study and designed the experiments. Xiaohuan Zhao wrote the manuscript. Xiaohuan Zhao, Mengqiao Xu and Zhenzhen Zhao performed the experiments with the help of Yang Liu, Yimin Wang and Yao Shen. Ting Zhang, Xiaoling Wan, Mei Jiang, Xueting Luo, Lei Chen, Minwen Zhou, Feng Wang and Xiaodong Sun interpreted data and contributed to discussion. All authors reviewed and concurred with the final manuscript. Xiaodong Sun and Feng Wang are the guarantor of this work and as such, had full access to all the data in the study and takes responsibility for the integrity of the data and the accuracy of the data analysis. All authors read and approved the final manuscript.

## FUNDING INFORMATION

This study was supported by grants from the National Natural Science Foundation of China (82171076, 82071852), Science and Technology Commission of Shanghai Municipality (20Z11900400), Shanghai Hospital Development Center (SHDC2020CR2040B, SHDC2020CR5014), the National Natural Science Foundation of China (81771739), the Program for Professor of Special Appointments (Eastern Scholar) at Shanghai Institutions of Higher Learning, the Top Young Talent Program of Shanghai, the Innovative Research Team of High‐level Local Universities in Shanghai, Shanghai Sailing Program (22YF1435400), and the National Natural Science Foundation of China (8220040737).

## CONFLICT OF INTEREST STATEMENT

The authors declare that they have no competing interests.

## CONSENT FOR PUBLICATION

All authors have read the manuscript and indicated consent for publication.

## Supporting information


Figure S1–S3

## Data Availability

All data generated or analyzed during this study are included in this published article and its supplementary information files.
