# Supplementary material for: Bifidobacterium promotes retinal ganglion cell survival by regulating the balance of retinal glial cells
Source: CNS Neurosci Ther. 2023 Mar 16;29(Suppl 1):146–60. doi: 10.1111/cns.14165 (PMC10314105; doi:10.1111/cns.14165)
Supplement: Supplementary file 1 — Figure S1–S3 [file CNS-29-146-s001.pdf]

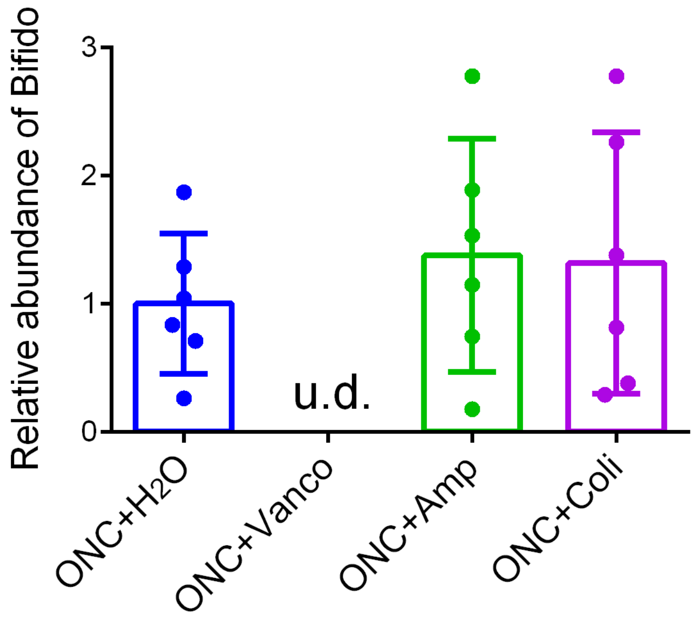

Supplementary Figure 1: The relative abundance of Bifidobacteria in the feces of mice treated with different antibiotics by RT-PCR. This was normalized to total bacteria (n=6, one-way ANOVA). u.d., undetectable.

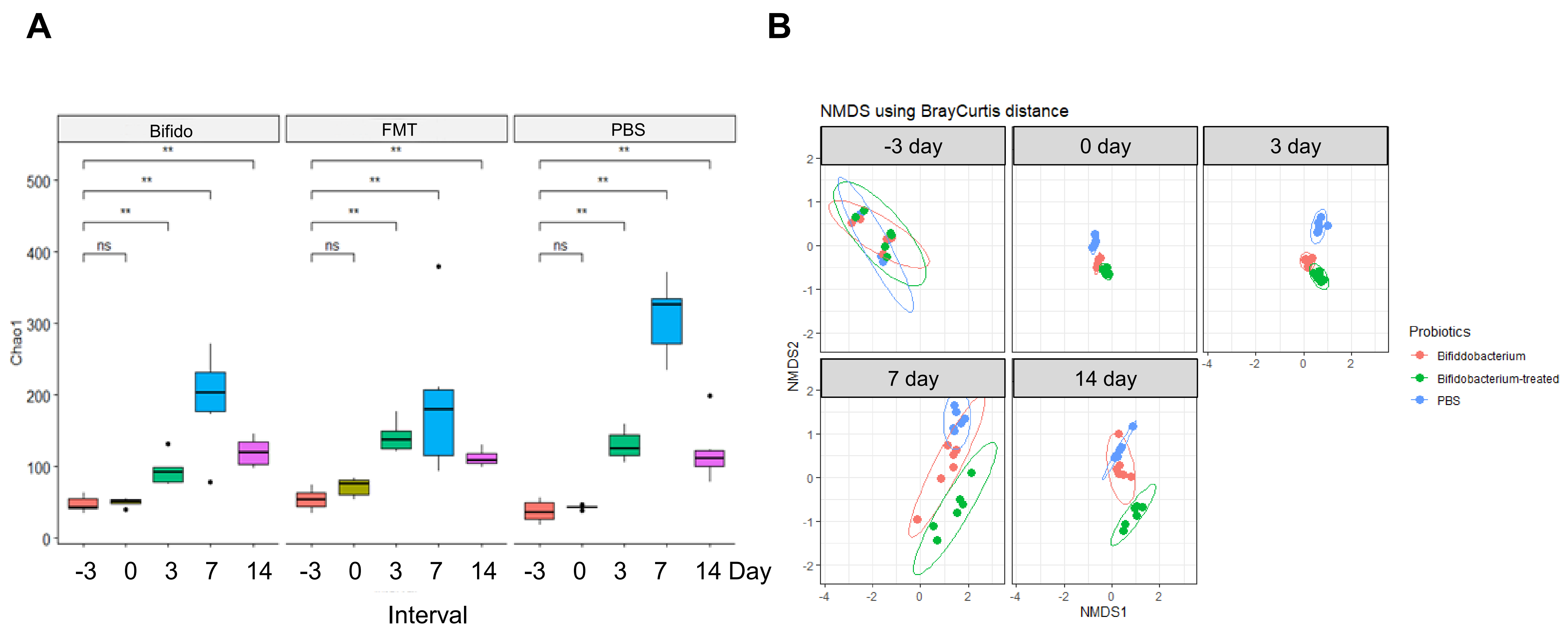

Supplementary Figure 2: α-Diversity and β-diversity based on the genus profile in three groups. **A.** α-Diversity (Chao1) based on the genus profile in three groups. **B.** β-diversity (BrayCurtis) based on the genus profile in three groups.

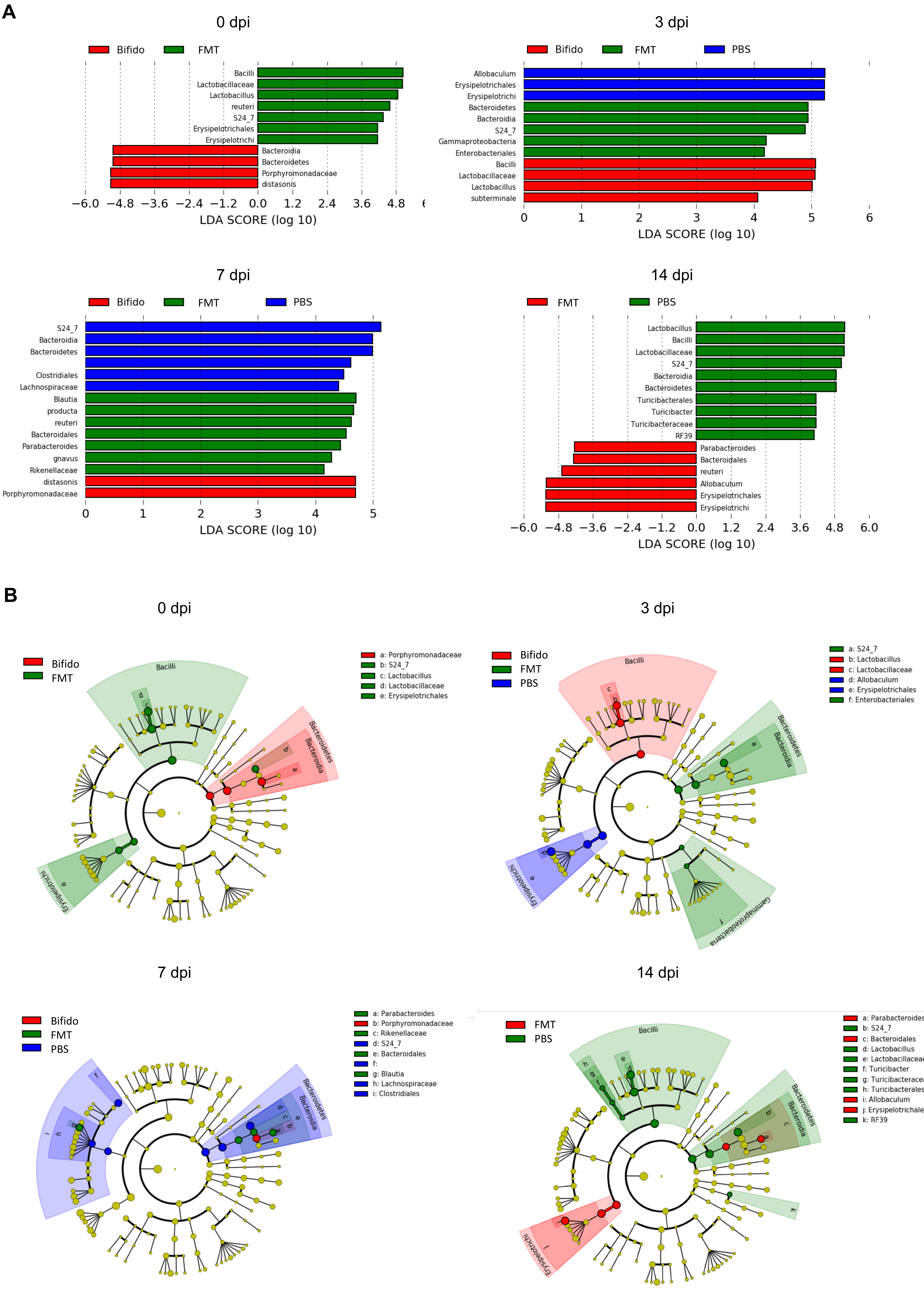
A
0 dpi
Bifido
FMT
Bacilli
Lactobacillaceae
Lactobacillus
reuteri
S24_7
Erysipelotrichales
Erysipelotrichi
Bacteroidia
Bacteroidetes
Porphyromonadaceae
distasonis
−6.0 −4.8 −3.6 −2.4 −1.2 0.0 1.2 2.4 3.6 4.8
LDA SCORE (log 10)
3 dpi
Bifido
FMT
PBS
Allobaculum
Erysipelotrichales
Erysipelotrichi
Bacteroidetes
Bacteroidia
S24_7
Gammaproteobacteria
Enterobacteriales
Bacilli
Lactobacillaceae
Lactobacillus
subterminale
0 1 2 3 4 5 6
LDA SCORE (log 10)
7 dpi
Bifido
FMT
PBS
S24_7
Bacteroidia
Bacteroidetes
Clostridiales
Lachnospiraceae
Blautia
producta
reuteri
Bacteroidales
Parabacteroides
gnavus
Rikenellaceae
distasonis
Porphyromonadaceae
0 1 2 3 4 5
LDA SCORE (log 10)
14 dpi
FMT
PBS
Lactobacillus
Bacilli
Lactobacillaceae
S24_7
Bacteroidia
Bacteroidetes
Turicibacterales
Turicibacter
Turicibacteraceae
RF39
Parabacteroides
Bacteroidales
reuteri
Allobaculum
Erysipelotrichales
Erysipelotrichi
−6.0 −4.8 −3.6 −2.4 −1.2 0.0 1.2 2.4 3.6 4.8 6.0
LDA SCORE (log 10)
B
0 dpi
Bifido
FMT
a: Porphyromonadaceae
b: S24_7
c: Lactobacillus
d: Lactobacillaceae
e: Erysipelotrichales
Bacilli
Bacteroidetes
Bacteroidia
Erysipelotrichi
3 dpi
Bifido
FMT
PBS
a: S24_7
b: Lactobacillus
c: Lactobacillaceae
d: Allobaculum
e: Erysipelotrichales
f: Enterobacteriales
Bacilli
Bacteroidetes
Bacteroidia
Erysipelotrichi
Gammaproteobacteria
7 dpi
Bifido
FMT
PBS
a: Parabacteroides
b: Porphyromonadaceae
c: Rikenellaceae
d: S24_7
e: Bacteroidales
f:
g: Blautia
h: Lachnospiraceae
i: Clostridiales
Bacteroidetes
Bacteroidia
14 dpi
FMT
PBS
a: Parabacteroides
b: S24_7
c: Bacteroidales
d: Lactobacillus
e: Lactobacillacea
f: Turicibacter
g: Turicibacterace
h: Turicibacterales
i: Allobaculum
j: Erysipelotrichale
k: RF39
Bacilli
Bacteroidetes
Bacteroidia
Erysipelotrichi

Supplementary Figure 3: Difference in the bacterial distribution among three groups. **A.** Comparative analysis of the microbial communities at the family, genus and species levels using the LEfSe method in three groups at different time. At 3 dpi, LDA scores (log10) for the most prevalent taxa in *Bifidobacterium* group are represented on the positive scale, whereas negative LDA scores indicate enriched taxa in *Bifidobacterium* group. At other times, positive LDA scores are presented among 3 groups. **B.** Cladograms of six different taxonomic levels (from phylum to genus). At 3 dpi, Green circles and shadings show the significantly enriched bacterial taxa obtained in *Bifidobcterium* group. Red circles and shadings show significantly enriched bacterial taxa obtained in *Bifido*-fecal group. At other times, Green circles and shadings show the significantly enriched bacterial taxa obtained in PBS group. Blue circles and shadings show significantly enriched bacterial taxa obtained in *Bifidobacterium* group. Red circles and shadings show significantly enriched bacterial taxa obtained in *Bifido*-fecal group.
